# *Akkermansia muciniphila* ameliorates the age-related decline in colonic mucus thickness and attenuates immune activation in accelerated aging *Ercc1*^*−/Δ7*^ mice

**DOI:** 10.1186/s12979-019-0145-z

**Published:** 2019-03-08

**Authors:** Benthe van der Lugt, Adriaan A. van Beek, Steven Aalvink, Ben Meijer, Bruno Sovran, Wilbert P. Vermeij, Renata M. C. Brandt, Willem M. de Vos, Huub F. J. Savelkoul, Wilma T. Steegenga, Clara Belzer

**Affiliations:** 1 Division of Human Nutrition and Health, Wageningen University and Research, Wageningen, The Netherlands; 2 Department of Immunology, Erasmus University Medical Center, Rotterdam, The Netherlands; 3 Cell Biology and Immunology Group, Wageningen University and Research, Wageningen, The Netherlands; 4 Laboratory of Microbiology, Wageningen University and Research, Wageningen, The Netherlands; 5 Host Microbe Interactomics, Wageningen University and Research, Wageningen, The Netherlands; 6 Department of Molecular Genetics, Erasmus University Medical Center, Rotterdam, The Netherlands; 7 Princess Máxima Center for Pediatric Oncology, Oncode Institute, Utrecht, The Netherlands; 8 Immunobiology Research Programme, Department of Bacteriology and Immunology, University of Helsinki, Helsinki, Finland

**Keywords:** Aging, *Akkermansia muciniphila*, Intestinal immunity, Mucus layer, Intestinal barrier

## Abstract

**Background:**

The use of *Akkermansia muciniphila* as potential therapeutic intervention is receiving increasing attention. Health benefits attributed to this bacterium include an improvement of metabolic disorders and exerting anti-inflammatory effects. The abundance of *A. muciniphila* is associated with a healthy gut in early mid- and later life. However, the effects of *A. muciniphila* on a decline in intestinal health during the aging process are not investigated yet. We supplemented accelerated aging *Ercc1*^*−/Δ7*^ mice with *A. muciniphila* for 10 weeks and investigated histological, transcriptional and immunological aspects of intestinal health.

**Results:**

The thickness of the colonic mucus layer increased about 3-fold after long-term *A. muciniphila* supplementation and was even significantly thicker compared to mice supplemented with *Lactobacillus plantarum* WCFS1. Colonic gene expression profiles pointed towards a decreased expression of genes and pathways related to inflammation and immune function, and suggested a decreased presence of B cells in colon. Total B cell frequencies in spleen and mesenteric lymph nodes were not altered after *A. muciniphila* supplementation. Mature and immature B cell frequencies in bone marrow were increased, whereas B cell precursors were unaffected. These findings implicate that B cell migration rather than production was affected by *A. muciniphila* supplementation. Gene expression profiles in ileum pointed toward a decrease in metabolic- and immune-related processes and antimicrobial peptide production after *A. muciniphila* supplementation. Besides, *A. muciniphila* decreased the frequency of activated CD80^+^CD273^−^ B cells in Peyer’s patches. Additionally, the increased numbers of peritoneal resident macrophages and a decrease in Ly6C^int^ monocyte frequencies in spleen and mesenteric lymph nodes add evidence for the potentially anti-inflammatory properties of *A. muciniphila*.

**Conclusions:**

Altogether, we show that supplementation with *A. muciniphila* prevented the age-related decline in thickness of the colonic mucus layer and attenuated inflammation and immune-related processes at old age. This study implies that *A. muciniphila* supplementation can contribute to a promotion of healthy aging.

**Electronic supplementary material:**

The online version of this article (10.1186/s12979-019-0145-z) contains supplementary material, which is available to authorized users.

## Background

Coincident with the increase in the aged population that is observed nowadays, the often inevitable decline in overall health in the elderly is becoming an alarming problem. The aging process is accompanied by a chronic low-grade inflammatory state, termed ‘inflamm-aging’, which is a strong risk factor for many age-related pathologies [1–4]. One of the organs that is affected by the aging process is the intestinal tract and the occurrence of gut-related disorders in the aged population is considerable [5].

As main inhabitant of the intestine, the gut microbiota play an essential role in the maintenance of overall health. Bacteria are able to degrade complex carbohydrates, thereby converting these substrates into metabolites that are beneficial to health, such as short-chain fatty acids (SCFAs) [6, 7]. Besides, the gut microbiota interact extensively with the host immune system by the regulation of immune responses [8]. During the aging process, changes in gut microbiota composition occur, such as a decreased diversity, a decrease in health-promoting bacteria and an increase in potential pathobionts. This disturbed balance in microbiota composition is thought to increase the risk of impaired intestinal barrier function and intestinal inflammation [9]. In mice, transfer of microbiota from aged mice to young germfree recipient mice promoted intestinal inflammation, increased leakage of bacterial components into blood and stimulated systemic immune activation [10].

An important factor with regard to gut health is the mucus layer that covers the intestinal epithelial cell layer and serves as physical protection for bacterial penetration and harmful compounds to enter the mucosal tissue [11]. Intestinal mucus is built up of mostly Mucin 2 (Muc2) proteins, which are large gel-forming proteins that are secreted by goblet cells located in the intestinal mucosa. These proteins form a net-like structure and are the building blocks of the mucus layer. The importance of the mucus layer was emphasized in studies using Muc2 knockout mice, which did not have a colonic mucus layer covering the intestinal epithelial layer [12, 13]. These mice suffered from a decreased intestinal barrier function, an increased inflammatory status [14] and had signs of colitis [12]. Next to the protective function of the mucus layer, it also serves as an energy source for bacteria. *Akkermansia muciniphila* is one of the bacterial species that is able to degrade mucus. This bacterium is highly abundant (~ 3%) in the healthy human colon [15]. Upon mucus degradation, *A. muciniphila* produces several immune-stimulating compounds, such as SCFAs and pili [16, 17]. The outer membrane pili-like protein Amuc_1100 is thought to be involved in the beneficial properties of *A. muciniphila* on health [18, 19].

Recent studies suggest that the beneficial effects of *A. muciniphila* are not limited to the intestinal tract, but extend to overall health. The abundance of *A. muciniphila* was reduced in people suffering from obesity, type 2 diabetes, inflammatory bowel disease, amongst others [20]. Furthermore, supplementation with *A. muciniphila* in mice resulted in an improved metabolic state and reduced diet-induced obesity (ClinicalTrials.gov Identifier: NCT02637115) [21–23].

We and others previously showed that the abundance of *Akkermansia* spp. in colonic luminal content decreased during aging in mice [10, 24, 25]. Another study also reported an age-related loss of *Akkermansia* spp. in humans [26]. Interestingly, the abundance of *Akkermansia* spp. was shown to be increased in centenarians (105–109 years old) compared to younger age groups [27]. These results could indicate that a relation exists between reaching an extreme old age and the abundance of *Akkermansia* spp. [24, 27].

The numerous potential beneficial characteristics of *A. muciniphila* suggest that this bacterium could be a potent candidate for microbial supplementation. However, the effects of this bacterium on the decline in intestinal health as seen during aging are not widely investigated yet. Therefore, the aim of the present study was to investigate the effects of supplementation with *A. muciniphila* on different aspects of intestinal health. We used *Ercc1*^*−/Δ7*^ mice, an accelerated aging mouse model that has a median lifespan of ~ 20 weeks. Further characteristics of this mouse model were extensively described in previous studies [28–30] and indicate that the accelerated aging phenotype of *Ercc1*^*−/Δ7*^ mice largely resembles normal aging. The *Ercc1*^*−/Δ7*^ mice were supplemented with *A. muciniphila* for 10 weeks via oral gavage. After sacrifice, ileum and colon were subject to transcriptional analysis and the microbiota composition in these organs was investigated. Furthermore, we assessed mucus thickness in the colon and the distribution of immune cells in immune-related tissues.

## Results

### *A. muciniphila* supplementation increased mucus thickness in the colon of *Ercc1*^*−/Δ7*^ mice

**Fig. 1:**
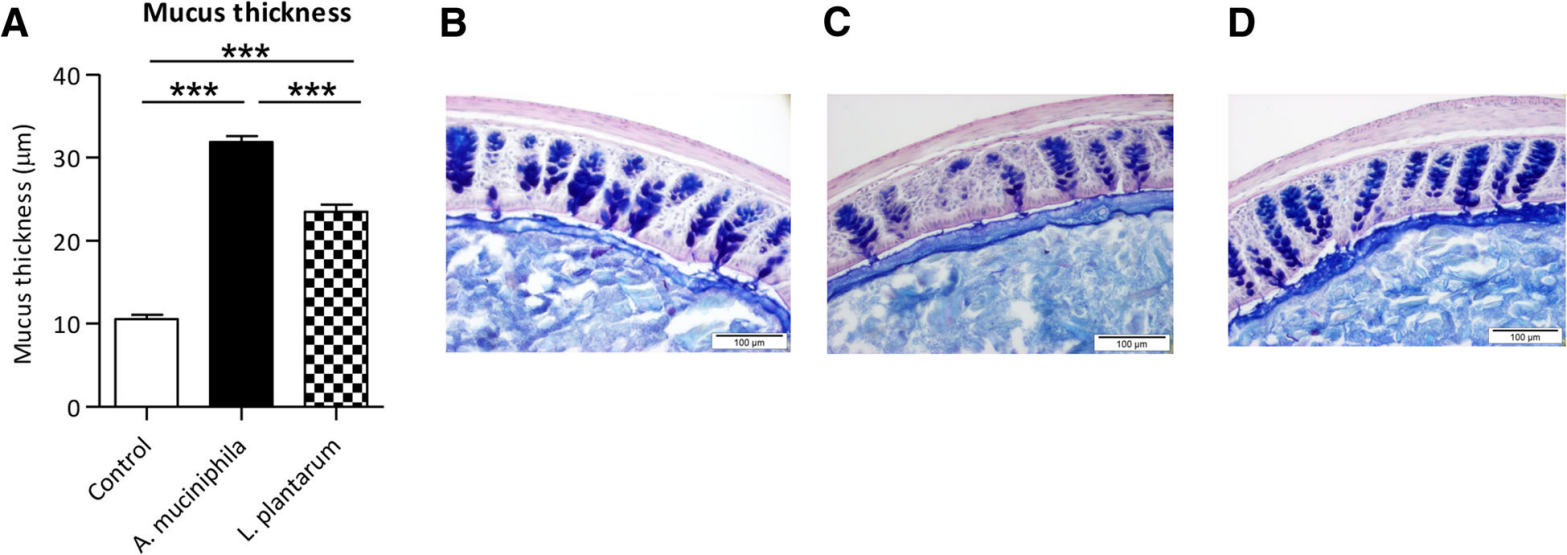
Mucus thickness increased in *Ercc1*^*−/Δ7*^ mice supplemented with *Akkermansia muciniphila*. **a** Mucus thickness (μm) measured in colon of *Ercc1*^*−/Δ7*^ mice in the control, *A. muciniphila* group and *L. plantarum* group. **b** Representative picture of PAS/Alcian Blue staining in control mouse, (**c**) mouse supplemented with *A. muciniphila* and (**d**) *L. plantarum*. Data represent the mean + SEM from three to five mice per group. ***p < 0.001. Scale bars histological images: 100 μm

Since *A. muciniphila* is a mucus-colonizing bacterium and utilizes mucus as energy source, we investigated whether supplementation with *A. muciniphila* had an effect on the mucus layer in the colon of *Ercc1*^*−/Δ7*^ mice. Measurements of mucus thickness in PAS/Alcian Blue stained colon tissue revealed that the mucus layer was significantly thicker in the mice supplemented with *A. muciniphila* compared to the control group (*p* < 0.001) (Fig. 1a-c). Besides, the results were compared with the mucus thickness of mice supplemented with *L. plantarum* (WCFS1), since we showed previously that supplementation with this bacterium prevented an age-related decline in mucus thickness [29]. The colonic mucus layer of *L. plantarum* supplemented mice was thicker compared to the control group (*p* < 0.001) (Fig. 1a, d), but supplementation with *A. muciniphila* resulted in a significantly thicker mucus layer than the *L. plantarum* supplemented mice (*p* < 0.001) (Fig. 1a). These results show that supplementation with *A. muciniphila* contributed to the prevention of a decreased mucus layer thickness at old age.

### No differences in colonic and ileal microbiota composition after supplementation with *A. muciniphila*

**Fig. 2:**
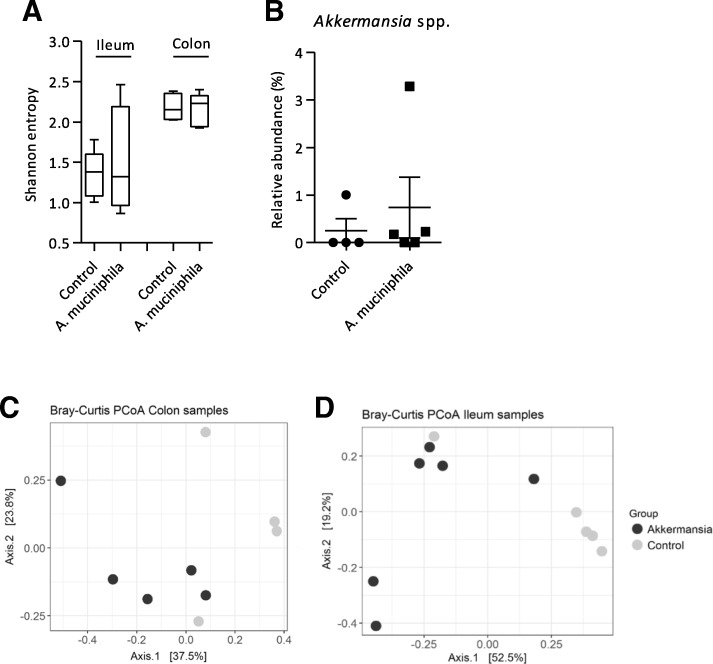
Microbiota composition in colon and ileum of *Ercc1*^*−/Δ7*^ mice supplemented with *Akkermansia muciniphila*. **a** Alpha-diversity (Shannon entropy) measured in ileum and colon samples. Boxes extend from the 25th to 75th percentile, middle line represents median, and whiskers represent minimum and maximum values. **b** Relative abundance (%) of *Akkermansia* spp. in colonic content assessed by 16S rRNA sequencing. Data represent mean + SEM (**c**) Beta-diversity measured by Bray-Curtis Principal Coordinate Analysis in colon samples and (**d**) ileum samples. Between four and six mice per group were used for microbiota analysis

In order to investigate whether supplementation with *A. muciniphila* caused changes in gut microbiota composition, we performed 16S rRNA gene sequencing on colonic and ileal content. Alpha-diversity (Shannon entropy) of colonic and ileal content samples did not differ between the control and supplemented mice (Fig. 2a). To investigate whether supplementation with *A. muciniphila* resulted in an increased colonization of this bacterium, the relative abundance of *Akkermansia* spp. in colonic content was assessed. In colonic content, the average relative abundance was slightly higher in the intervention group (0.738 ± 1.279%) compared to the control group (0.252 ± 0.503%) (Fig. 2b), but this difference was not statistically significant. *Akkermansia* spp. was not present in ileal content, except for one mouse in the intervention group (data not shown). Variation in microbial composition between samples was represented in a principal coordinates analysis (PCoA) based on Bray-Curtis dissimilarity. In both colon and ileum samples, no clear separation was observed between the control and *A. muciniphila* group (Fig. 2c-d). Furthermore, no statistically significant differences at genus level between the control and supplemented mice was found in both sources (data not shown). These data show that bacterial supplementation with *A. muciniphila* three times a week did not result in changes in gut microbiota composition of *Ercc1*^*−/Δ7*^ mice.

### *A. muciniphila* supplementation minimally altered the expression of genes involved in intestinal barrier function in colon

To explore the effects of supplementation with *A. muciniphila* on gene expression, transcriptome analysis was performed on mRNA isolated from colon and ileum tissue of *Ercc1*^*−/Δ7*^ mice. In colon, a number of 427 genes was significantly differentially expressed (*p* < 0.05, fold change > 1.2 or < − 1.2) between the control and *A. muciniphila* group, comprising 225 up-regulated and 202 down-regulated genes. Since a highly significant increase in mucus thickness was observed in the colon of mice that received *A. muciniphila*, colonic expression of genes related to mucus production was investigated. No significant differential expression of mucins was observed, except Mucin like 1 (*Mucl1*) which was down-regulated (− 1.5-fold) in the *A. muciniphila* group compared to the control mice (Additional file 1). Besides, to explore if an increased mucus thickness in the colon resulted in an enhanced intestinal barrier function, expression of genes related to tight junction function was investigated. The classical tight junction proteins *Tjp 1–3*, *Jam 1–3*, *Claudin* family and *Ocln* were not differentially expressed (data not shown). To investigate which pathways were regulated in colon by *A. muciniphila* supplementation, Gene Set Enrichment Analysis (GSEA) was performed. Significantly enriched pathways were dominated by cell cycle related processes, but no pathways related to intestinal barrier function were observed (Additional file 2).

### Supplementation with *A. muciniphila* decreased expression of genes and pathways related to antimicrobial activities, metabolic processes and mucus biosynthesis in ileum

**Fig. 3:**
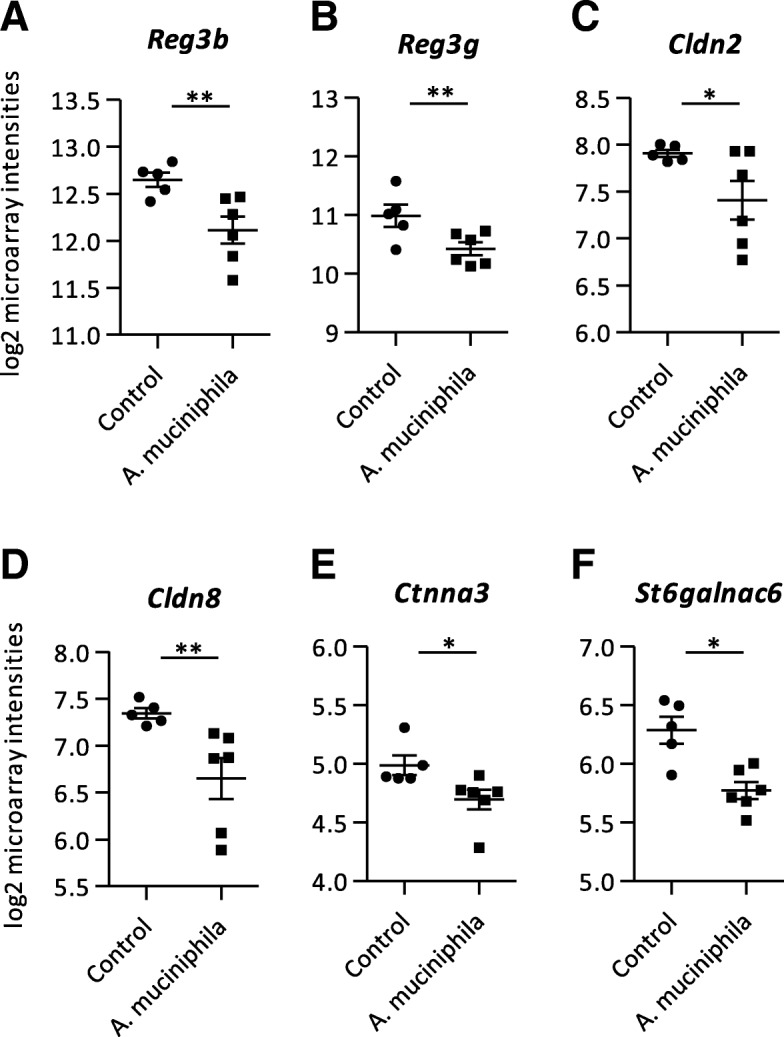
Microarray analysis performed on mRNA isolated from ileum tissue. **a** Log2 microarray intensities of Regenerating islet-derived 3 beta (*Reg3b*), (**b**) Regenerating islet-derived 3 gamma (*Reg3g*) (**c**) Claudin 2 (*Cldn2*), (**d**) Claudin 8 (*Cldn8*), (**e**) Catenin (cadherin associated protein), alpha 3 (*Ctnna3*) and (**f**) ST6 (alpha-N-acetyl-neuraminyl-2,3-beta-galactosyl-1,3)-N-acetylgalactosaminide alpha-2,6-sialyltransferase 6 (*St6galnac6*). Data represent mean + SEM. Control: *n* = 5, *A. muciniphila*: *n* = 6. *p < 0.05; **p < 0.01

Since supplementation with *A. muciniphila* minimally altered the expression of genes related to intestinal barrier function in colon, we also investigated gene expression profiles in ileum tissue of *Ercc1*^*−/Δ7*^ mice. A number of 795 genes was significantly differentially expressed (*p* < 0.05, fold change > 1.2 or < − 1.2) between the control and *A. muciniphila* group in ileum, comprising 425 up-regulated and 370 down-regulated genes. Interestingly, several genes encoding for antimicrobial peptides were down-regulated in the *A. muciniphila* supplemented mice, i.e. *Reg3b* and *Reg3g* (Fig. 3a, b; Additional file 3). However, the expression of genes encoding for alpha-defensins and lysozymes was not affected by *A. muciniphila* supplementation. Tight junction genes were minimally differentially expressed, only *Cldn2* and *Cldn8* were down-regulated in ileum of mice supplemented with *A. muciniphila* compared to the control group (Fig. 3c, d; Additional file 3). GSEA revealed that significantly enriched down-regulated pathways were dominated by metabolic processes (Additional file 2). Besides, the pathways “N-Glycan Biosynthesis” and “Biosynthesis of the N-Glycan Precursor (Dolichol Lipid-Linked Oligosaccharide, LLO) and Transfer to a Nascent Protein” were down-regulated in ileum of mice that received *A. muciniphila* supplementation compared to the control group (Additional file 2). Based on this finding, the microarray data set was searched for genes related to mucus biosynthesis. The genes *Ctnna3* and *St6galnac6* were down-regulated in ileum in the supplemented mice versus control group (Fig. 3e, f; Additional file 3).

### Decreased expression of genes and pathways related to inflammation and immune function in colon and ileum after *A. muciniphila* supplementation

**Fig. 4:**
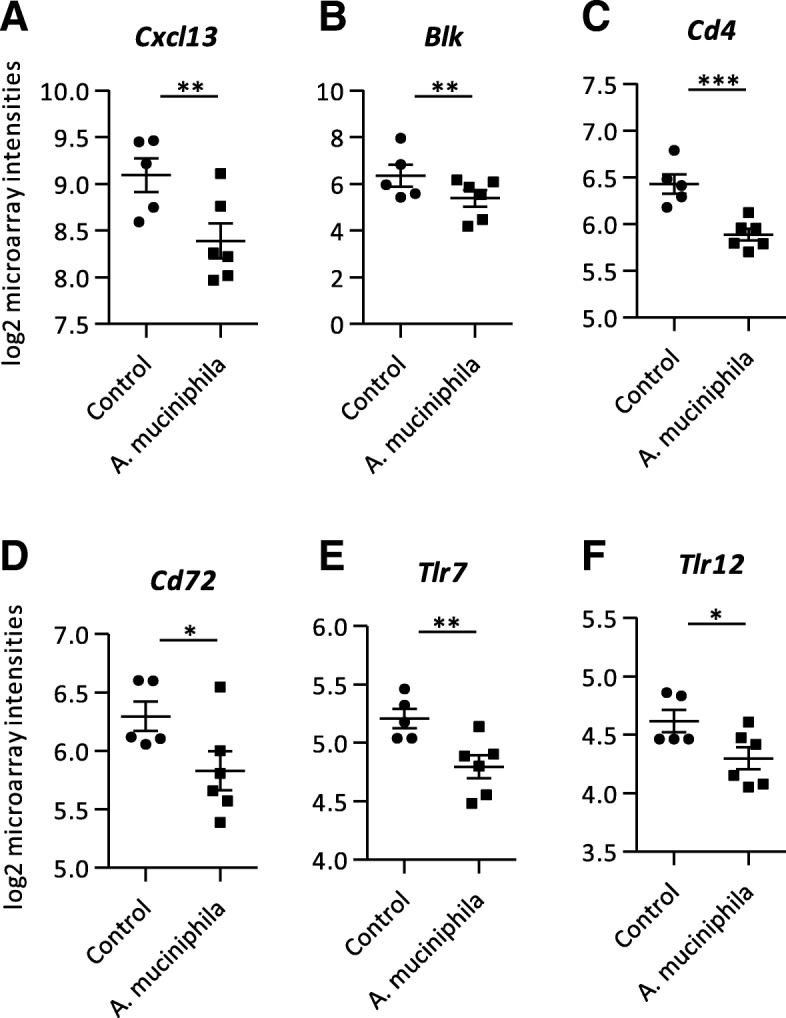
Microarray analysis performed on mRNA isolated from colon tissue. **a** Log2 microarray intensities of (**a**) C-X-C motif chemokine ligand 13 (*Cxcl13*), (**b**) B lymphoid kinase (*Blk*), (**c**) Cluster of differentiation 4 (*Cd4*), (**d**) Cluster of differentiation 72 (*Cd72*), (**e**) Toll-like receptor 7 (*Tlr7*), (**f**) Toll-like receptor 12 (*Tlr12*). Data represent mean + SEM. Control group: *n* = 5. *A. muciniphila* group: *n* = 6. *p < 0.05; **p < 0.01; ***p < 0.001

As immune function is an important factor regarding intestinal health, expression profiles of genes related to immune response were investigated. Remarkably, down-regulated genes in both colon and ileum were dominated by genes encoding for immunoglobulins (Additional file 1). In the colon, several genes encoding for chemokines, such as *Cxcl13* (Fig. 4a; Additional file 3) and *Ccl12*, as well as the cytokine *Il5* and the complement factors *C1ra* and *C5ar1* were all down-regulated (Additional file 1). Also the immunoglobulin receptor *Pilrb1* had a decreased expression in the colon of the *A. muciniphila* group (Additional file 1). Additionally, other immune-related genes were down-regulated in the colon of *A. muciniphila* supplemented mice compared to the control group, e.g. *Blk*, *Cd4*, *Cd72*, *Tlr7* and *Tlr12* (Fig. 4b-f; Additional file 3). GSEA revealed that immune-related pathways were down-regulated in colon, for example “Intestinal Immune Network for IgA Production”, “Cytokine-Cytokine Receptor Interaction” and “Inflammatory Response Pathway”, amongst others (Additional file 2). Moreover, Ingenuity pathway analysis (IPA) identified seven cytokines as upstream regulators that were predicted to be inhibited after supplementation with *A. muciniphila*, including both the pro-inflammatory Il1 and anti-inflammatory Tgf-beta (Table 1). Also other inflammation-related factors, such as Myeloid differentiation primary response 88 (Myd88), Tumor necrosis factor receptor superfamily member 1B (Tnfrsf1b) and 12 (Tnfsf12), NFKB Inhibitor Alpha (Nfkbia), T cell receptor (TCR) and Toll Like Receptor Adaptor Molecule 1 (Ticam1) were predicted inhibited upstream regulators (Table 1). In ileum, GSEA annotated the pathway “Antigen Presentation: Folding, assembly and peptide loading of class I MHC” as highly up-regulated (Additional file 2). The most significant upstream regulator identified by IPA in ileum tissue was Interleukin 10 Receptor Subunit Alpha (Il10RA), which was predicted to be slightly inhibited after *A. muciniphila* supplementation (Table 2). Taken together, these results show that *A. muciniphila* supplementation decreased the expression of numerous genes and pathways related to inflammation and immune function in both colon and ileum.

**Table 1:** Upstream regulators in colon identified by Ingenuity pathway analysis based on the comparison between *Ercc1*^*−/Δ7*^ mice receiving *A. muciniphila* supplementation and control mice

Upstream Regulator	Activation z-score	*p*-value of overlap
ACOX1	2.24	0.013
Alpha catenin	2.00	0.029
ID3	1.98	0.030
**SOCS1**	1.95	0.040
CDKN2A	1.72	0.025
**MYD88**	−2.41	0.045
**IL1**	−2.38	0.049
**TNFRSF1B**	−2.22	0.003
**CHUK**	−2.21	0.017
GATA6	−2.21	0.036
**NFKBIA**	−2.17	0.029
Akt	−2.16	0.020
**cytokine**	−1.99	0.000
**Nfat (family)**	−1.98	0.014
CCND1	−1.98	0.044
**WNT5A**	−1.95	0.033
CTNNB1	−1.95	0.037
**Tgf beta**	−1.94	0.010
EGR1	−1.91	0.035
**Interferon alpha**	−1.78	0.004
**LTBR**	−1.72	0.001
**STAT3**	−1.71	0.024
**TCR**	−1.60	0.029
**TNFSF12**	−1.58	0.004
**TNF**	−1.54	0.036
SSB	−1.52	0.000
**IL17A**	−1.40	0.002
**TICAM1**	−1.40	0.003
HRAS	−1.39	0.031
E2F1	−1.36	0.009
**IL6**	−1.34	0.005
PRKACA	−1.21	0.001
**IL1B**	−1.21	0.003

Cut-off values include *p* < 0.05 and activation z-score < − 1.2 or > 1.2. Upstream regulators in bold are involved in inflammation- and immune-related processes

**Table 2:** Upstream regulators in ileum identified by Ingenuity pathway analysis based on the comparison between *Ercc1*^*-/Δ7*^ mice receiving *A. muciniphila* supplementation and control mice

Upstream Regulator	Activation z-score	*p*-value of overlap
HMGA1	1.66	0.002
POR	1.46	0.000
SYVN1	1.34	0.006
CFTR	1.25	0.000
AR	−2.74	0.005
NR1I2	−2.00	0.042
BRCA1	−1.98	0.016
CTNNB1	−1.72	0.001
LEP	−1.60	0.031
**MAPK14**	−1.34	0.020
**IL10RA**	−1.31	0.000

Cut-off values include *p* < 0.05 and activation z-score < − 1.2 or > 1.2. Upstream regulators in bold are involved in inflammation- and immune-related processes

### Minor changes in local B cell distribution after *A. muciniphila* supplementation

**Fig. 5:**
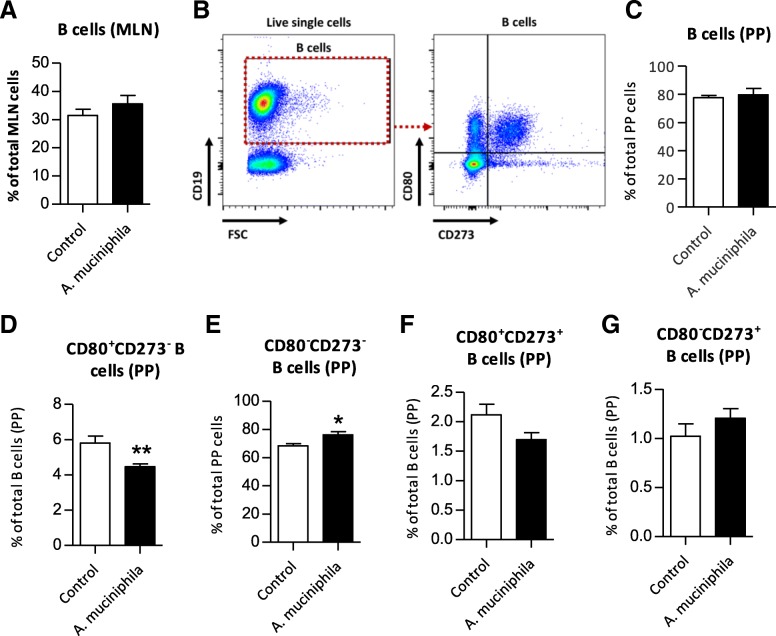
Distribution of B cell subsets in mesenteric lymph nodes (MLN) and Peyer’s patches (PP) after supplementation with *Akkermansia muciniphila*. **a** Mean frequency of B cells in MLN. **b** Flow cytometric analysis of B cells in PP. **c** Mean frequency of B cells in PP. **d** Mean frequency of CD80^+^CD273^−^ B cells, (**e**) CD80^−^CD273^−^ B cells, (**f**) CD80^+^CD273^+^ B cells and **g** CD80^−^CD273^+^ B cells in PP. Data represent the mean + SEM from five to six mice per group. *p < 0.05; **p < 0.01

Based on the findings of the microarray analysis, we continued with the investigation of immune cell distribution in different organs of the immune system. Since several B cell related genes were down-regulated after *A. muciniphila* supplementation, such as immunoglobulin genes (both in ileum and colon), and *Blk* (only in colon), we first focused on the local distribution of B cells. In mesenteric lymph nodes (MLN) and Peyer’s patches (PP), no differences in B cell frequencies between groups were observed (Fig. 5a-c). However, in PP the frequency of activated CD80^+^CD273^−^ B cells was significantly lower in the supplemented mice (*p* = 0.009) (Fig. 5d). Furthermore, frequencies of more immature CD80^−^CD273^−^ B cells were significantly higher in the *A. muciniphila* group (*p* = 0.03) (Fig. 5e), whereas no changes were observed in CD80^+^CD273^+^ memory-like and CD80^−^CD273^+^ B cells (Fig. 5f, g) [31].

### *A. muciniphila* supplementation increased the migration of B cells into spleen and BM

**Fig. 6:**
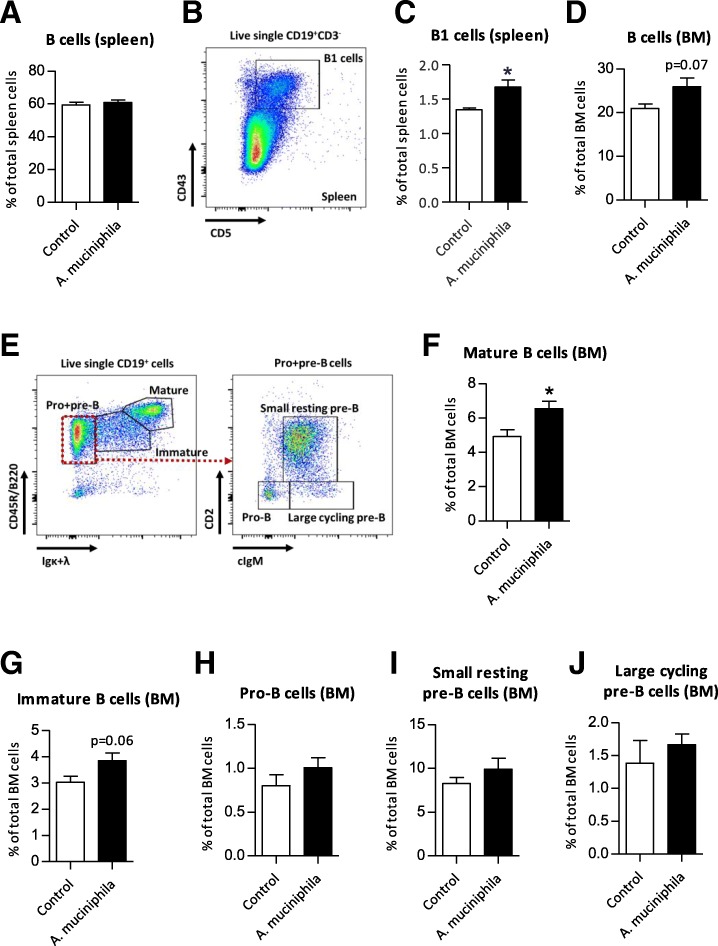
Distribution of B cell subtypes in spleen and bone marrow (BM) after supplementation with *Akkermansia muciniphila*. **a** Mean frequencies of CD19^+^CD3^−^ B cells in spleen. **b** Flow cytometric analysis of live single CD19^+^CD3^−^ cells in spleen. **c** Mean frequencies of CD5^+^CD43^+^ B1 cells in spleen. **d** Mean frequencies of B cells in BM. **e** Flow cytometric analysis of live single CD19^+^ cells, divided in pro-B cells, large cycling pre-B cells and small resting pre-B cells in BM. **f** Mean frequencies Mature B cells, **g** Immature B cells, **h** Pro-B cells, **i** Small resting pre-B cells and (**j**) Large cycling pre-B cells in BM. Data represent the mean + SEM from four to six mice per group. *p < 0.05

Next, we continued with investigation of B cell subsets in spleen and bone marrow (BM). The frequency of total B cells in spleen was not different between groups (Fig. 6a). Also other B cell subsets in spleen, such as immature, follicular and marginal zone B cells, were not significantly different between groups (data not shown). However, the frequency of B1 cells in spleen was significantly higher in the *A. muciniphila* supplemented mice (*p* = 0.02) (Fig. 6b, c). In bone marrow (BM), a trend of higher frequencies of total B cells was observed in *A. muciniphila* supplemented mice compared to control mice (*p* = 0.07) and frequencies of mature and immature B cells were also higher (p = 0.02 and 0.06, respectively) (Fig. 6d-g). Frequencies of B cell precursors, i.e. pro-B cells, small resting pre-B cells and large cycling pre-B cells were not different between groups (Fig. 6h-j). These data suggest that *A. muciniphila* supplementation did not change production of new B cells in BM, but increased migration of B cells into the spleen and BM.

### T cell distribution in MLN and spleen was unaltered after *A. muciniphila* supplementation

**Fig. 7:**
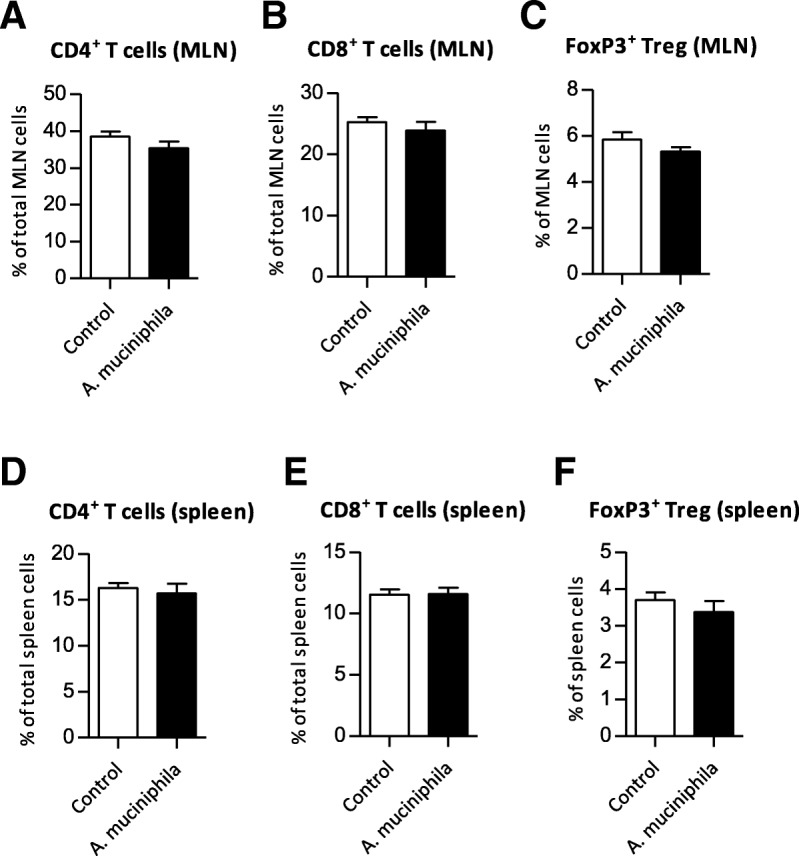
Distribution of T cells in mesenteric lymph nodes (MLN) and spleen after supplementation with *Akkermansia muciniphila*. **a** Mean frequencies of CD4^+^ T cells and (**b**) CD8^+^ T cells and (**c**) FoxP3^+^ Treg cells in MLN. **d** Mean frequencies of CD4^+^ T cells and (**e**) CD8^+^ T cells and (**f**) FoxP3^+^ Treg cells in spleen. Data represent the mean + SEM from five to six mice per group

Transcriptome analysis showed that expression of the *Cd4* gene was decreased in the colon of mice that received *A. muciniphila* compared to the control mice. Moreover, IPA revealed T cell receptor (TCR) as predicted inhibited upstream regulator in colon. Therefore, we investigated whether the distribution of T cells was altered between groups in spleen and MLN. However, CD4^+^ and CD8^+^ T cell distributions in MLN and spleen were not different compared to the control group, neither were CD4^+^FoxP3^+^ Treg frequencies changed in both immune tissues (Fig. 7a-f).

### Decreased inflammatory cell populations in spleen and MLN after *A. muciniphila* supplementation

**Fig. 8:**
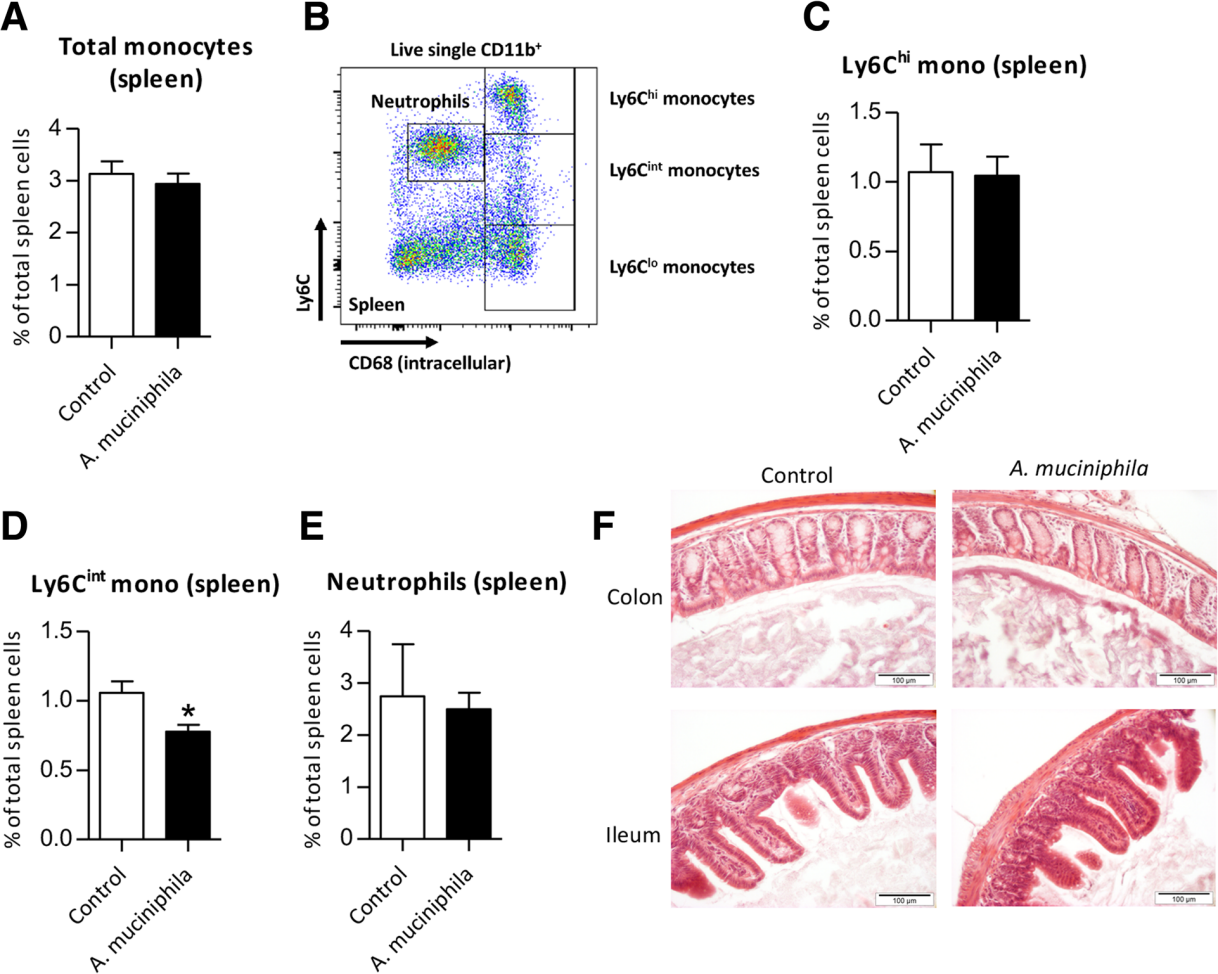
Distribution of inflammatory cell frequencies in spleen after supplementation with *Akkermansia muciniphila*. **a** Mean frequencies of total CD11b^+^CD68^+^ monocytes. **b** Flow cytometric analysis of live single CD11b^+^ Ly6C/CD68 cells, divided in Ly6C^hi^, Ly6C^int^, Ly6C^lo^ monocytes and CD68^dim^Ly6C^int/hi^ neutrophils. **c** Mean frequencies of Ly6C^hi^monocytes, (**d**) Ly6C^int^ monocytes and (**e**) neutrophils. (**f**) Representative pictures of H&E stained colon and ileum tissue of control and *A. muciniphila* supplemented mice. Data represent the mean + SEM from five to six mice per group. *p < 0.05

Next, since GSEA revealed an enrichment of pathways related to inflammatory response and immune function, we investigated whether inflammatory cell frequencies were altered after supplementation of *Ercc1*^*−/Δ7*^ mice. In spleen, the frequencies of total and Ly6C^hi^ monocytes were slightly lower in the *A. muciniphila* group and Ly6C^int^ monocytes were significantly lower (*p* = 0.01) (Fig. 8a-d). Besides, the frequency of neutrophils was also slightly lower in the supplemented mice (Fig. 8e). The same trends of these inflammatory cell populations were found in MLN, although cell frequencies were low (< 1%, data not shown). These data reveal a minor decrease in inflammatory markers in spleen and MLN after *A. muciniphila* supplementation in *Ercc1*^*−/Δ7*^mice. In addition, we assessed if we could identify any signs of immune cell infiltration by investigating H&E stained tissue. For both colon and ileum tissue, no histological signs of immune cell infiltration were observed in the control and *A. muciniphila* group (Fig. 8f).

### Increased numbers of CD11b^+^ cells and resident macrophages in peritoneum of *Ercc1*^*−/Δ7*^ mice supplemented with *A. muciniphila*

**Fig. 9:**
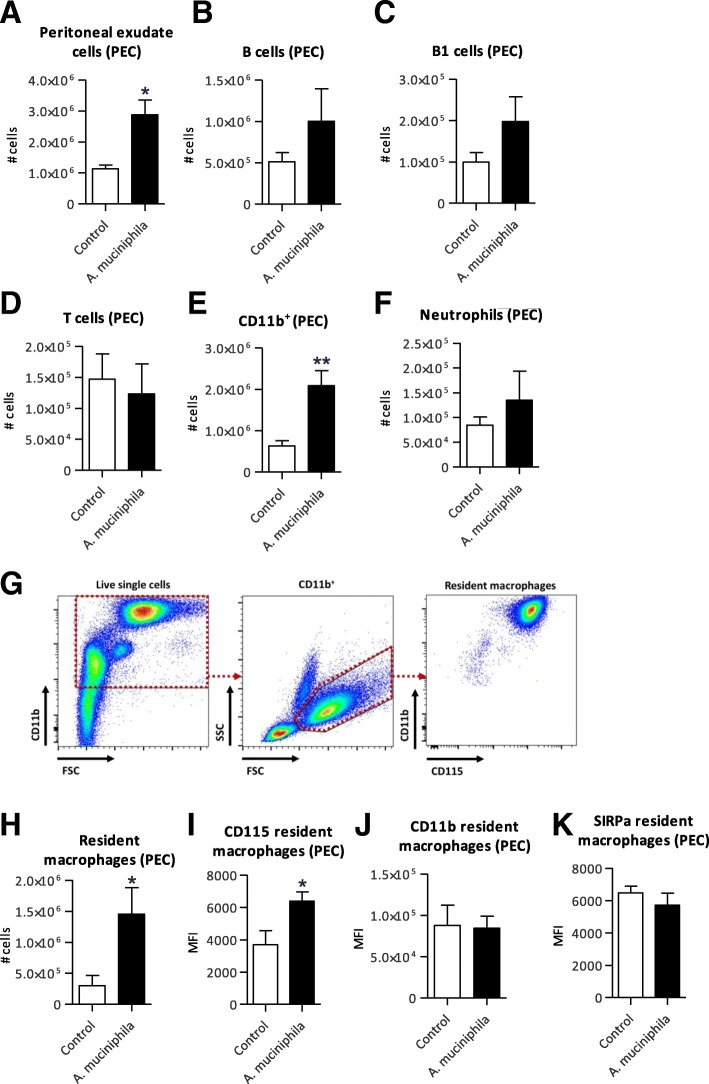
Distribution of immune cells in the peritoneum after supplementation with *Akkermansia muciniphila*. **a** Absolute number of total peritoneal exudate cells (PEC), (**b**) CD3^−^CD19^+^ B cells, (**c**) CD5^+^CD43^+^ B1 cells and (**d**) CD3^+^ T cells, (**e**) CD11b^+^ cells and (**f**) CD11b^+^Ly6G^+^ neutrophils. **g** Flow cytometric analysis of CD11b^+^ cells. **h** Absolute numbers of CD11b^+^Ly6G^−^ resident macrophages in peritoneal exudate cell suspension. **i** Median Fluorescence Intensity (MFI) of CD115 marker, (**j**) CD11b marker and (**k**) SIRPa marker expressed on resident macrophages. Data represent the mean + SEM from five to six mice per group. *p < 0.05; **p < 0.01

Since we found a higher abundance of B1 cells in spleen and this cell type is generally highly enriched in the peritoneal cavity, we also investigated the distribution of immune cells in the peritoneum. Remarkably, the total number of peritoneal cells in mice supplemented with *A. muciniphila* was nearly 3-fold higher than in the control mice (*p* = 0.02) (Fig. 9a). The absolute numbers of B cells, B1 cells and T cells did not significantly differ between groups (Fig. 9b-d). We found an increase in absolute numbers of CD11b^+^ cells in the peritoneum after *A. muciniphila* supplementation (*p* = 0.004) (Fig. 9e). Investigation of CD11b^+^ cell subsets revealed that absolute numbers of neutrophils were not significantly higher after *A. muciniphila* supplementation (Fig. 9f), in contrast to resident macrophages (*p* = 0.045) (Fig. 9g, h). In addition, the expression of CD115 on resident macrophages was significantly higher in the *A. muciniphila* group compared to the control group (*p* = 0.02) (Fig. 9i), whereas no change in the expression of CD11b and SIRPα was observed (Fig. 9j, k).

### *A. muciniphila* supplementation did not alter survival, body weight and organ weights of *Ercc1*^*−/Δ7*^ mice

Supplementation with *A. muciniphila* for 10 weeks did not alter survival rates as compared to the control mice (Additional file 4). Body weight of *Ercc1*^*−/Δ7*^ mice increased in the first half of life, but decreased again from 11 weeks of age onwards (Additional file  4). No pronounced differences in body weight development were found between groups. The weight of liver, spleen and thymus measured directly after sacrifice were not significantly different between control and *A. muciniphila* group (data not shown).

## Discussion

The bacterium *Akkermansia muciniphila* is suggested to be a promising microbial supplement, due to its beneficial effects on health [20, 32]. However, its effects on the decline in intestinal health during aging are not well investigated yet. In the present study, we investigated the effects of supplementation with *A. muciniphila* on different aspects of intestinal health in accelerated aging *Ercc1*^*−/Δ7*^ mice. We report that supplementation with *A. muciniphila* for 10 weeks resulted in a significantly thicker colonic mucus layer and an improvement of anti-inflammatory immune status compared to the control group.

In two recent studies, it has been shown that the colonic mucus layer decreased in aging mice, suggesting an association with bacterial penetration and immune activation [29, 33]. An aging-related decrease in mucus thickness was also observed in *Ercc1*^*−/Δ7*^ mice [29], confirming the similarity in aging phenotype in this accelerated aging mouse model compared to a mouse model with a normal aging process. We previously showed that *L. plantarum* WCFS1 increased the thickness of the colonic mucus layer in *Ercc1*^*−/Δ7*^ mice [29], but we now show that *A. muciniphila* is capable to even further thicken the mucus layer. The ability of *A. muciniphila* to increase mucus thickness was also reported before [23]. *A. muciniphila* is able to degrade mucin structures to use it as carbon and nitrogen source and is therefore called a mucus-degrader [17]. Nevertheless, the observed increased mucus layer thickness after supplementation suggests that this bacterium is able to actively turn on host colonic mucus production, a suggestion that has also been made by Derrien and colleagues [34]. Interestingly, we did not find differential expression of any genes encoding for mucins, apart from a down-regulation of *Mucl1* in colon tissue. However, expression levels of this gene were low and it is not a typical mucin that constitutes the colonic mucus layer, such as *Muc2* [35]. In our previous study, supplementation with *L. plantarum* WCFS1 did neither result in differences in mucin gene expression, while a significantly increased colonic mucus thickness was observed [29]. Possibly, supplementation with *A. muciniphila* resulted in an increased mucus thickness by impacting mucus biosynthesis processes without affecting *Muc2* expression. In order to understand the exact underlying mechanisms of the mucus turnover processes, further investigation is warranted.

Interestingly, in the ileum of *A. muciniphila* supplemented mice, GSEA revealed a down-regulation of two pathways related to N-Glycan biosynthesis and besides, two genes related to mucus biosynthesis were also down-regulated. These results imply that, besides its great impact on the colonic mucus layer, *A. muciniphila* could also have had an effect on the ileal mucus layer. We could not verify this finding by measuring the ileal mucus layer, because of its rather discontinuous appearance due to the presence of villi.

Next to the important function of the mucus layer, tight junctions (TJs) sealing the intestinal epithelial cells also play an important role in intestinal barrier function and an age-related decreased expression of TJ genes was found in baboons [36]. We and others previously showed that *A. muciniphila* improved intestinal barrier function in a Caco-2 cell model [37] as well as in mice [18, 19, 23, 38, 39]. In the present study, we found a down-regulation of *Cldn2* and *Cldn8* in the ileum of *A. muciniphila* supplemented mice. However, the intestinal barrier consists of a complex structure of multiple protein networks [40]. Therefore, it is not possible to draw any solid conclusions on the effects of *A. muciniphila* supplementation on intestinal barrier function based on gene expression data only.

Gut microbiota composition and bacterial diversity in ileum and colon were not significantly changed after supplementation with *A. muciniphila*. This result indicates that bacterial supplementation with *A. muciniphila* does not lead to a reshaped gut microbiota composition, which was also reported before [23]. Gut microbiota analysis revealed that the relative abundance of *A. muciniphila* was low in ileal and colonic content. It was previously shown that *Akkermansia* spp. was more present in colon than ileum in mono-colonised mice [39], but the low abundance in colon was remarkable. The dose of the bacterium, i.e. 2 × 10^8^ CFU for 3 times a week, was already proven effective in previous mice studies [23]. Possibly, the relatively long time between the last oral gavage and sacrifice (about 24 h) could have led to a washout of *A. muciniphila*. Though, a recent study showed that daily supplementation with 11 probiotic strains resulted in low colonization in mice which was caused by the indigenous microbiome [41]. Possibly, this finding may also explain the impeded colonization of *A. muciniphila* in our study.

Bacterial supplementation with *A. muciniphila* resulted in a down-regulation of numerous immune-related genes and pathways in colon. Notably, these included several B cell related genes, such as immunoglobulins, *Blk* and *Pilrb1*, amongst others. Moreover, GSEA revealed a down-regulation of the pathway “Intestinal immune network for IgA production”. These results imply that *A. muciniphila* supplementation may have decreased the necessity for producing IgA, i.e. exerting a mucosal protective reaction against commensal bacteria [42]. In line with these results, we found a down-regulation of both *Tlr7* and *Tlr12* in colon. However, we could not confirm this hypothesis, since IgA concentrations in colon were not measured during this study. We did investigate B cell frequencies in several immune tissues. Nevertheless, we could not find differences in B cell frequencies in MLNs, whereas B cell frequencies were increased in spleen and BM. These findings suggest that based on transcriptome analysis, *A. muciniphila* decreased B cell frequencies and CD4^+^ T cells in colon and caused a potential redistribution of B and T cells among lymphoid organs. Conversely, frequencies of total B cell subsets in BM and spleen were slightly increased after *A. muciniphila* supplementation, with no change in B cell precursor frequencies in BM. These findings may indicate that supplementation with *A. muciniphila* inhibited influx of B cells into the colon, leading to a slightly increased mature B cell pool in spleen and BM.

Furthermore, we found a decrease in inflammatory markers in colon after *A. muciniphila* supplementation. Several genes encoding for chemokines, complement factors, as well as the cytokine *Il5* were down-regulated after *A. muciniphila* supplementation. Besides, IPA identified numerous pro-inflammatory cytokines as potentially inhibited upstream regulators in colon. The anti-inflammatory properties of *A. muciniphila* are already extensively described [34]. We now add evidence that *A. muciniphila* might protect against the aging-related increase in inflammation (inflamm-aging) by decreasing the colonic expression of pro-inflammatory genes and pathways. Besides, we also identified the anti-inflammatory cytokine Tgf-beta as inhibited upstream regulator. Although we only found one anti-inflammatory cytokine, this finding may point toward a general reduction of immune activation by *A. muciniphila*. Histological analysis of the colon did not reveal any clear signs of immune infiltration in both groups. In a previous study by Derrien and colleagues, an up-regulation of immune related genes with no signs of microscopically visible inflamed tissue was also observed in mice mono-colonized with *A. muciniphila* [39]. The authors suggest that these observations were part of regulatory processes of immune tolerance toward *A. muciniphila*. However, in this study germ-free mice were used, hence any comparisons between these particular results and our results should be made with caution.

Interestingly, we found significantly lower frequencies of activated B cell subtypes and higher frequencies of more immature B cell subtypes in PP. The increased level of inactive immature B cells is in accordance with the microarray results from ileum tissue, since we also found decreased expression of numerous immunoglobulin-related genes. Besides, *Reg3b* and *Reg3g* were both down-regulated in ileum. We previously showed that *A. muciniphila* supplementation also decreased *Reg3g* expression in ileum of mice fed a high-fat diet [19]. In a recent study, an increased expression of antimicrobial genes was found in the ileum of aged C57BL/6 mice, including *Reg3b*, *Reg3g*, *Defb1* and *Retnlb*, which was suggested to be related to an increased state of epithelial distress [43]. Hence, based on our findings we suggest that supplementation with *A. muciniphila* might contribute to prevention of the age-related state of epithelial distress in ileum.

It is well-known that T cell function declines during the aging process [44]. Our previous study revealed that supplementation with *L. plantarum* WCFS1 and *L. casei* BL23 increased regulatory T cell frequencies in MLN of *Ercc1*^*−/Δ7*^ mice. However, supplementation with *A. muciniphila* did not lead to any changes in T cell distribution in MLN, spleen and PP, despite a down-regulation of the *Cd4* gene in colon and the predicted inhibition of the upstream regulator TCR. Possibly, the increased colonic mucus layer caused by *A. muciniphila* supplementation resulted in an increased protective state in the colon, thereby decreasing the production of a number of T cell attracting chemokines and subsequently leading to a decrease of CD4^+^ T cell attraction and infiltration.

In the peritoneal cavity, a highly significant increase in resident macrophages was observed after *A. muciniphila* supplementation. Peritoneal macrophages are important in the modulation of immune responses during infections and contribute to tissue homeostasis [45]. Furthermore, peritoneal resident macrophages were shown to defend against microbial invasion [46], which could explain the high presence of this cell type after supplementation with *A. muciniphila*. However, this increase in peritoneal resident macrophages was not coincided with increased frequencies of neutrophils and T cells, while these cell types are expected to be highly present during inflammation. This observation implies that supplementation with *A. muciniphila* resulted in an activated state with regard to peritoneal resident macrophages, but resulted in an anti-inflammatory rather than a pro-inflammatory response.

## Conclusions

The attention for *A. muciniphila* as a potential microbial supplement has increased and ample evidence exists emphasising the beneficial effects of this bacterium on low-grade inflammation and (cardio)metabolic disorders [16, 20, 34, 47]. In the present study, we also observed that several metabolic processes in ileum, as well as immunological processes in both ileum and colon were affected by *A. muciniphila*, but now in an aging model. Furthermore, we convincingly showed that *A. muciniphila* has a protective effect against an age-related decline in mucus thickness, which was even stronger compared to *L. plantarum* WCFS1. Aging is often accompanied by low-grade inflammation and an increased risk on metabolic syndrome [1, 48], contributing to a decreased quality of life and a considerable rise in healthcare costs [49]. Our study showed a causal relationship between *A. muciniphila* and attenuation of the aging phenotype, in terms of preventing the age-related decline in thickness of the colonic mucus layer and improving immune status. These results therefore support the therapeutic application of *A. muciniphila* in the aging population and pave the way for further studies investigating *A. muciniphila* as therapeutic intervention contributing to healthy aging. Further research should focus on the practical aspects for application in humans, such as the dosage, frequency and way of administration.

## Materials and methods

### Mice and study design

In this study, accelerated aging *Ercc1*^*−/Δ7*^ mice were used. Genotyping of this mouse model was extensively described previously by others [28–30]. In short, *Ercc1*^*−/Δ7*^ mice have an impaired DNA repair protein ERCC1, resulting in accumulation of a broad variety of DNA lesions and consequently accelerated aging. Mice were individually housed under SPF conditions, received an ad libitum purified diet (formula D12450B, Research Diets, Additional file 5a) and had ad libitum access to water supplied by water bottles with long nozzles. Mice were supplemented with *Akkermansia muciniphila* Muc^T^ (ATTC BAA-835) by oral gavage at a dose of 2 × 10^8^ CFU/200 μL, three times a week, for a total of 10 weeks. Oral gavages were given in the morning. The control group simultaneously received oral gavages containing the same volume of PBS. A third *Ercc1*^*−/Δ7*^ mice group was included that received the same dose of *Lactobacillus plantarum* WCFS1. These mice were only used for histological purposes. Growing procedures of the bacterial cultures was extensively described previously [29]. A number of 18 mice per group (both male and female) was included and lifespan of these mice was assessed. After 10 weeks, when the mice were 16 weeks old, a number of 5–6 female mice were sacrificed. Colonic and ileal content, as well as distal ileum and proximal colon sections, were collected and snap-frozen in liquid nitrogen. A piece of ileal and colonic tissue was fixed in Carnoy’s solution for histological purposes. Spleen, mesenteric lymph nodes, Peyer’s patches, bone marrow and peritoneal exudate cells were isolated for immunological measurements.

### Histology

After paraffin embedding, Carnoy-fixed distal ileum and proximal colon tissue were sliced in 5 μM sections on poly-l-lysine coated glass slides (Thermo Scientific, Germany). Slides were dewaxed, dehydrated and stained with hematoxylin and eosin (H&E) and PAS/Alcian blue. The thickness of the colonic mucus layer was measured using ImageJ software (NIH, MD, USA). For comparison of the mucus layer thickness, we included an extra mouse group that received *Lactobacillus plantarum* WCFS1.

### Microbiota composition analysis

DNA was isolated from ileal and colonic content using a modified repeated bead beating method [50]. Microbiota composition was assessed using 16S rRNA sequencing on the MiSeq platform (Illumina, San Diego, CA, USA). Next, the NG-Tax pipeline was used for barcode-primer filtering, de-multiplexing, OTU picking and taxonomic classification [51]. The generated biom-files were used for summarizing the microbiological data, i.e. alpha-diversity and beta-diversity, using the R-packages microbiome [52] and phyloseq [53].

### RNA isolation

RNA was isolated from distal ileum and proximal colon tissue (*n* = 5–6 mice/group) using TRIzol reagent (Invitrogen, Breda, The Netherlands). Purification of the isolated RNA was performed using the RNeasy Mini kit (Qiagen, Venlo, The Netherlands). After measurement of the total RNA yield (Nanodrop, ND-1000, Nanodrop Products, Maarssen, The Netherlands), RNA integrity was assessed (Agilent 2100 Bioanalyzer, Agilent Technologies, Amsterdam, The Netherlands) and only RNAs were included with a RNA integrity number (RIN) above 8.0.

### Microarray analysis

Microarray analysis was performed as described previously [54]. Differences in gene expression between the control and *A. muciniphila* supplemented mice groups were assessed using the Intensity Based Moderated T statistics (IBMT) method, with *p*-values < 0.05 and fold changes <− 1.2 or > 1.2. Microarray data has been submitted to the NCBI Gene Expression Omnibus (GEO) (GSE126730). Gene Set Enrichment Analysis (GSEA) was used to identify significantly enriched pathways [55]. Only pathways with a False Discovery Rate (FDR) < 0.2 were taken into consideration. Ingenuity pathway analysis (IPA) was used for the identification of upstream regulators [56].

### cDNA synthesis and real-time quantitative PCR

Real-time quantitative PCR (qPCR) was used to validate the expression profiles of a selection of differentially expressed genes identified in the microarray analysis. For both colon and ileum samples, complementary DNA (cDNA) was synthesized from 1000 ng of total RNA using the RevertAid First Strand cDNA Synthesis Kit (Thermo-Fisher Scientific, Landsmeer, The Netherlands) following the manufacturer’s protocol. The following thermal cycling conditions were used: 5 min at 25 °C, 60 min at 37 °C and 5 min at 70 °C. Primer sequences were obtained at the online PrimerBank database (Additional file 5b) [57]. qPCR was performed with a CFX384 thermal cycler (Bio-Rad Laboratories, Veenendaal, the Netherlands) using the SensiMix SYBR No-ROX kit (Bioline, Alphen aan den Rijn, The Netherlands). The housekeeping gene 36B4 was used for normalization.

### Fluorescence-activated cell sorting (FACS) analysis

Spleen, mesenteric lymph nodes, Peyer’s patches, bone marrow and peritoneal exudate cells were all subject to FACS analysis, similar as previously reported [29]. In brief, femurs, tibias, ileac crests, forelegs, and sternum were harvested and crushed with mortar and pestle. Singe-cell suspensions from each organ were prepared by passing cells through a 40-μm cell strainer with a syringe. First, cells were stained for extracellular markers. Fixable live/dead eFluor506 stain (Ebioscience, San Diego, USA) was used to exclude dead cells. Cells were fixed and permeabilized with Fix/Perm buffer (Ebioscience) to stain for intracellular markers. All antibodies used for flow cytometry are enlisted in Additional file 5c. A Canto II flow cytometer was used (BD Biosciences, Erembodegem, Belgium) and data analysis was performed using FlowJo vX.07 software (Tree Star Inc., USA).

### Statistical analysis

The Kolmogorov-Smirnov test was used to test if data were normally distributed and appropriate non-parametric statistical tests were used when data were not normally distributed. With regard to the survival analysis, the log-rank (Mantel-Cox) test was used. To test differences between the control and supplemented group, a student t-test or Mann-Whitney U test was performed. With regard to the differences in mucus thickness, a Kruskal-Wallis test with Dunn’s multiple comparisons test was performed, since three mouse groups were involved. Unless otherwise stated, *p*-value levels of *p* < 0.05 were considered as statistically significant.

## Additional files

Additional file 1: Gene expression profiles (Microarray analysis) of colon and ileum. Only genes that were statistically significantly different (*p* < 0.05) and had a fold change (FC) < − 1.2 or > 1.2 between groups are included. (XLSX 64 kb)

Additional file 2: Gene set enrichment analysis (GSEA) results of ileum and colon. Only significantly enriched pathways (FDR < 0.2) are included. (XLSX 25 kb)

Additional file 3: Relative gene expression of (A) Regenerating islet-derived 3 beta (*Reg3b*), (B) Regenerating islet-derived 3 gamma (*Reg3g*), (C) Claudin 2 (*Cldn2*), (D) Claudin 8 (*Cldn8*), (E) Catenin (cadherin associated protein), alpha 3 (*Ctnna3*) and (F) ST6 (alpha-N-acetyl-neuraminyl-2,3-beta-galactosyl-1,3)-N-acetylgalactosaminide alpha-2,6-sialyltransferase 6 (*St6galnac6*) in ileum. (G) Relative expression of C-X-C motif chemokine ligand 13 (*Cxcl13*), (H) B lymphoid kinase (*Blk*), (I) Cluster of differentiation 4 (*Cd4*), (J) Cluster of differentiation 72 (*Cd72*), (K) Toll-like receptor 7 (*Tlr7*), (L) Toll-like receptor 12 (*Tlr12*) in colon. (PDF 266 kb)

Additional file 4: Survival rates and body weight of *Ercc1*^*−/Δ7*^ mice. (A) Percent survival of all mice. These data include 12–13 mice per group with an additional 5–6 per group censored at 16 weeks. (B) Percentage survival of only male mice (*n* = 8–10/group) and (C) female mice (*n* = 8–10/group). (D) Body weight in grams measured weekly in all mice (*n* = 18 mice per group), (E) male mice (n = 8–10/group) and (F) female mice (n = 8–10/group). NB: A number of 5–6 female mice was sacrificed at 16 weeks. (PDF 261 kb)

Additional file 5: (A) Table with diet composition. (B) Table with primer sequences used for qPCR. (C) Table with a list of antibodies used in flow cytometry. (DOCX 21 kb)

## Abbreviations

BM: Bone marrow
CFU: Colony-forming unit
*Ercc1*^*-/Δ7*^ mice: mice with defective nucleotide excision repair gene Ercc1
FACS: Fluorescence-activated cell sorting
FC: Fold change
FDR: False discovery rate
GSEA: Gene set enrichment analysis
H&E: Hematoxylin and eosin
IBMT: Intensity based moderated T statistics
IPA: Ingenuity pathway analysis
MLN: Mesenteric lymph node
PAS: Periodic acid-Schiff
PCoA: Principal coordinates analysis
PP: Peyer’s patch
qPCR: Quantitative polymerase chain reaction
SPF: Specific pathogen-free
TJ: Tight junction
