# Supplementary figures and images for: Akkermansia muciniphila ameliorates the age-related decline in colonic mucus thickness and attenuates immune activation in accelerated aging Ercc1−/Δ7 mice
Source: Immun Ageing. 2019 Mar 8;16:6. doi: 10.1186/s12979-019-0145-z (PMC6408808; doi:10.1186/s12979-019-0145-z)

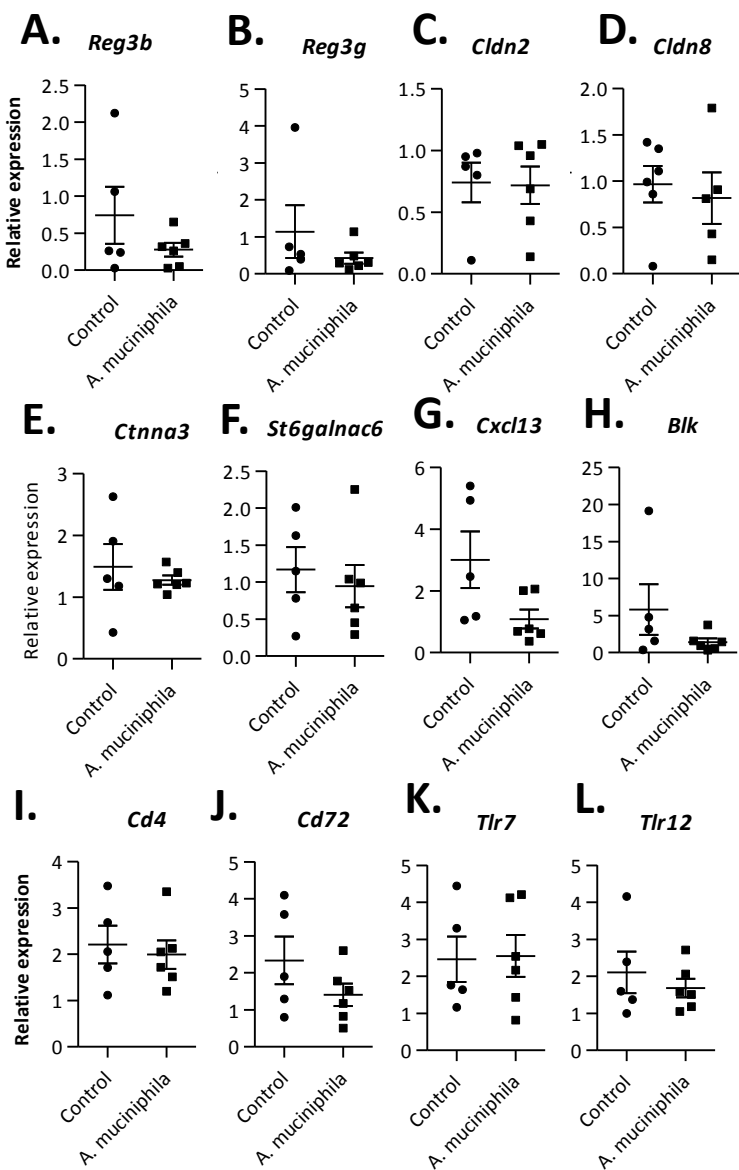

Supplement: Supplementary file 3 — Relative gene expression of (A) Regenerating islet-derived 3 beta (Reg3b), (B) Regenerating islet-derived 3 gamma (Reg3g), (C) Claudin 2 (Cldn2), (D) Claudin 8 (Cldn8), (E) Catenin (cadherin associated protein), alpha 3 (Ctnna3) and (F) ST6 (alpha-N-acetyl-neuraminyl-2,3-beta-galactosyl-1,3)-N-acetylgalactosaminide alpha-2,6-sialyltransferase 6 (St6galnac6) in ileum. (G) Relative expression of C-X-C motif chemokine ligand 13 (Cxcl13), (H) B lymphoid kinase (Blk), (I) Cluster of differentiation 4 (Cd4), (J) Cluster of differentiation 72 (Cd72), (K) Toll-like receptor 7 (Tlr7), (L) Toll-like receptor 12 (Tlr12) in colon. (PDF 266 kb) [file 12979_2019_145_MOESM3_ESM.pdf]

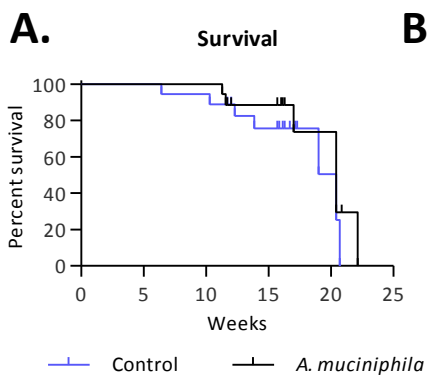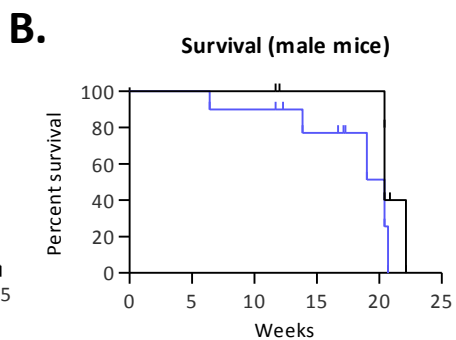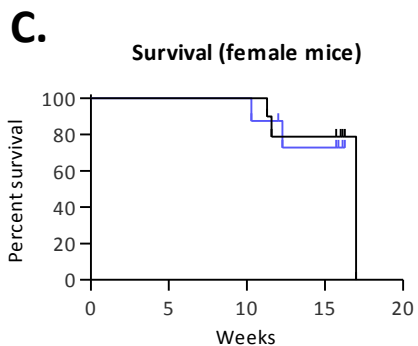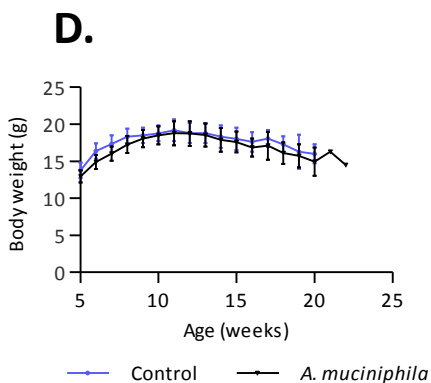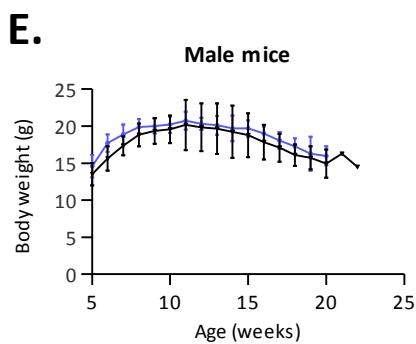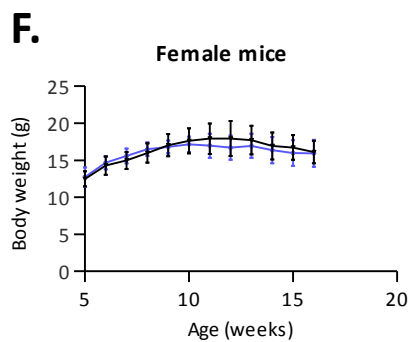

Supplement: Supplementary file 4 — Survival rates and body weight of Ercc1−/Δ7 mice. (A) Percent survival of all mice. These data include 12–13 mice per group with an additional 5–6 per group censored at 16 weeks. (B) Percentage survival of only male mice (n = 8–10/group) and (C) female mice (n = 8–10/group). (D) Body weight in grams measured weekly in all mice (n = 18 mice per group), (E) male mice (n = 8–10/group) and (F) female mice (n = 8–10/group). NB: A number of 5–6 female mice was sacrificed at 16 weeks. (PDF 261 kb) [file 12979_2019_145_MOESM4_ESM.pdf]
