# Supplementary material for: Akkermansia muciniphila ameliorates the age-related decline in colonic mucus thickness and attenuates immune activation in accelerated aging Ercc1−/Δ7 mice
Source: Immun Ageing. 2019 Mar 8;16:6. doi: 10.1186/s12979-019-0145-z (PMC6408808; doi:10.1186/s12979-019-0145-z)
Supplement: Supplementary file 5 — (A) Table with diet composition. (B) Table with primer sequences used for qPCR. (C) Table with a list of antibodies used in flow cytometry. (DOCX 21 kb) [file 12979_2019_145_MOESM5_ESM.docx]

**Additional file 5A** Composition of the D12450B purified diet.

| **Based on formula # D12450B** |  | |
| --- | --- | --- |
|  | **gm%** | ***kcal%*** |
| Protein | 19 | *20* |
| Carbohydrate | 67 | *69* |
| Fat | 4 | *10* |
| Other | 10 | *1* |
| Total | 100 | *100* |
| kcal/gm | 3.8 |  |
|  |  |  |
| **Ingredient** | **gm** | ***kcal*** |
| Casein, lactic | 200 | *800* |
| L-Cystine | 3 | *12* |
|  |  |  |
| Corn Starch | 427.2 | *1709* |
| Maltodextrin | 100 | *400* |
| Sucrose | 172.8 | *691* |
|  |  |  |
| Cellulose, BW200 | 50 | *0* |
|  |  |  |
| Soybean oil | 25 | *225* |
| Palm oil | 20 | *180* |
|  |  |  |
| Mineral Mix S10026* | 10 | *0* |
| DiCalcium Phosphate | 13 | *0* |
| Calcium Carbonate | 5.5 | *0* |
| Potassium Citrate, 1 H2O | 16.5 | *0* |
|  |  |  |
| Vitamin Mix V10001* | 10 | *40* |
| Choline chloride | 2 | *0* |
|  |  |  |
| **Total** | **1055** | ***4057*** |

**Additional file 5B** List of all primer sequences that were used for qPCR.

| **Gene Name** | **Forward primer (5’ → 3’)** | **Reverse primer (5’ → 3’)** |
| --- | --- | --- |
| *Reg3b* | ACTCCCTGAAGAATATACCCTCC | CGCTATTGAGCACAGATACGAG |
| *Reg3g* | AGGCCCTCAGGACATCTTGT | ATAGCCCAGTGTCGGGTCAT |
| *Cldn2* | CAACTGGTGGGCTACATCCTA | CCCTTGGAAAAGCCAACCG |
| *Cldn8* | GCAACCTACGCTCTTCAAATGG | TTCCCAGCGGTTCTCAAACAC |
| *Ctnna3* | AAGAATGGCCGAGTCAAGGAA | GCAGCATTTATGATCTGTGGACA |
| *St6galnac6* | AACAGTGCCAACGAGGTCTTC | CTTGTTGCCGAGGATAGGGAA |
| *Cxcl13* | GGCCACGGTATTCTGGAAGC | GGGCGTAACTTGAATCCGATCTA |
| *Blk* | GAGGCAGGTCAGTGAGAAGG | GTCCTGGTTAGGAGATGGTGG |
| *Cd4* | TCCTAGCTGTCACTCAAGGGA | TCAGAGAACTTCCAGGTGAAGA |
| *Cd72* | GCTCAGGGAGAAGATAAGTCAGC | GCGTCCTCGTGAGTCCTCT |
| *Tlr7* | CACCACCAATCTTACCCTTACC | CAGATGGTTCAGCCTACGGAA |
| *Tlr12* | TTGGAAGTTGTACCTCGGACT | GAAGTTGGGTAAGGTGCAGAC |
| *36B4* | ATGGGTACAAGCGCGTCCTG | GCCTTGACCTTTTCAGTAAG |

**Additional file 5C** List of all antibodies used in flow cytometry.

| Target | Format | Clone | Company |
| --- | --- | --- | --- |
| CD2 | PE | RM2-5 | BD |
| CD3e | PerCP-Cy5.5 | 145-2C11 | Ebioscience |
| CD4 | APC-H7 | GK1.5 | BD |
| CD5 | APC | 53-7.3 | BD |
| CD8a | PerCP-Cy5.5 | 53-6.7 | BD |
| CD11b | APC-Cy7  BV421  PE-Cy7  PerCP-Cy5.5 | M1/70 | BD  BD  Ebioscience  Ebioscience |
| CD16/32 | Purified | 2.4G2 | BD |
| CD19 | APC-eFluor780  PerCP-Cy5.5 | 1D3 | Ebioscience |
| CD43 | PE | S11 | BioLegend |
| CD45R/B220 | BV421 | RA3-6B2 | BD |
| CD68 | FITC | FA-11 | BioLegend |
| CD80 | BV421 | 16-10A1 | BD |
| CD115 | PE | AFS98 | Ebioscience |
| CD172a/SIRPα | APC | P84 | Ebioscience |
| CD273/PDL2 | PE | TY25 | BD |
| FoxP3 | PE | FJK-16s | Ebioscience |
| IgD | PE-Cy7 | 11-26c | Ebioscience |
| Igκ | FITC | 187.1 | BD |
| Igλ | FITC | R26-46 | BD |
| IgM | APC | II/41 | Ebioscience |
| Ly6C | AF488  PerCP-Cy5.5 | ER-MP20  HK1.4 | AbD Serotec  Ebioscience |
| Ly6G | PE-Cy7 | 1A8 | BD |
